# Pathological Anatomy

**Published:** 1836-04-01

**Authors:** 


### WORKS ON PATHOLOGICAL ANATOMY.

I. Outlines of Human Pathology. By *Herbert Mayo*, F.R.S. &c. &c. Part I. 8vo.

II. Illustrations of the Elementary Forms of Disease. By *Robert Carswell*, M.D. &c. Fasciculus Ninth. Hypertrophy.

[Continued from No. 47, page 34.]

If our readers will refer to the pathological article contained in the las number of this Journal, they will find that we abruptly quitted Mr. Mayo and postponed the further notice of his work until the present quarter. We concluded the review of the morbid changes of the bones, and joints, and bursæ, and we stated that the tissues whose alterations remained to be considered, were the muscular, the nervous, and cutaneous. To those our attention is now to be directed.

We have urged so repeatedly our conviction of the benefits to be derived from the diffusion of a knowledge of pathology, that we will not repeat the trite and hackneyed reasons that have led to that conclusion. But correct ideas on pathological subjects are not the offspring of the closet. The Editor of a Journal calculated to disseminate sound practical opinions must not now be sought in some country-town. *He* may indeed learn what others do, and, neglecting that knowledge which lives and breathes, instruct us in the fantasies of Gallic ingenuity, or plunge us in the gloom of German mysticism. He may shew the inquisitive practitioner how little the treatment of disease is understood in some great or some small Continental city, and display still more nakedly the well-known fact, that the fruits of Continental medicine and the triumphs of Continental professors must be sought almost solely in the dead-house and dissecting-room. All this may be done by industrious pedantry, and the book-learned journalist may suppose that he is contributing to the advancement of his art.

But the man who is to live by curing diseases speedily discovers the hollowness of this. He seeks in vain for some useful fact, some truths that he may apply in the moments of difficulty and distress. Those truths are not found in the subtle meshes of transcendental criticism, or in the flimsy web of eunuch dissertations, too general for medicine, and too medical for general science. The utilitarian glances with contempt at the emasculated page, and seeks in the simple study of facts the sterling knowledge that *it* cannot give.

For ourselves we are content to rest our claims for support on uninterrupted and undeviating efforts to put down what is hypothetical and vague, and to foster the spirit of inductive reasoning. It is induction, carried to the uttermost, that alone can extricate medical science from the alluvies of conjecture and uncertainty, in which it has been left by the successive deluges of systematic theorists from Hippocrates to Cullen. On morbid anatomy our induction must be founded—but that is only the foundation. Those who think that morbid anatomy has done all it can do, are undoubtedly deceived. The alterations of many tissues are yet imperfectly investigated—the earlier phenomena of morbid changes have hitherto been almost unexamined—the chemistry of morbid products is all but a blank page. It is absurd to say that we have arrived at the ultima thule of pathology. We have not all nor nearly all its primary facts—the great conclusions are yet to come. When they *do* come, there is still a wide field of analysis almost unbroken. Then, and not till then, shall we be enabled to decide what medicine can and cannot compass, then, and not before, can we really determine what our remedial experiments are worth. The great object at present is to cultivate morbid anatomy, and obtain an exact acquaintance with the data that it furnishes.

But unfortunately those data are too frequently presented encumbered with error, or invested with far more importance than they merit. It is here that sound criticism is of value. Such criticism must be the offspring of experience, and that experience can only be obtained where the opportunities of pathological inquiries are great. The provincial critic can scarcely have enjoyed them, and he probably retails the sentiments of others. In the last few years more than one attempt has been made to erect the tribunal of medical criticism in the Provinces. It has hitherto universally failed, and the same ill-success will probably continue to attend it. It would not be difficult to display the disadvantages under which a medical periodical must labour, when generated in the fields. Those fields are not the fields of science, which thrives in the busy haunts of men. Physic, like Dr. Johnson, loves " the full tide of human existence in Charing Cross." Her offspring are most lusty amidst the smoke of cities—her rural births are usually abortive. But we must return to Mr. Mayo.

Affections of the muscles and their tendons occupy a short chapter of eight pages. Yet there are some circumstances connected with the pathology of muscles, that claim the consideration of the practical surgeon and physician.

Mr. Mayo thus divides the study of diseases of the muscles:—he investigates separately the changes of their proper structure—and the morbid phenomena which result from the influence of the nervous supply they receive from the brain and spinal cord. Reparation after injury, hypertrophy, atrophy, inflammation, and malignant disease, are comprised under the first head; palsy, anaesthesia, muscular neuralgia, and spasm, under the second.

The phenomena of reparation after injuries really exhibit no peculiarity. But Mr. Mayo makes one or two practical observations, which are highly deserving of attention. Surgeons would appear, for what reason it is difficult to conjecture, to entertain a horror of dividing muscles. In operations, particularly on the arteries, they will go out of their way to avoid cutting a muscular fibre, and dodge about in all directions, rather than inflict a wound upon it. To be sure, the margin of a muscle frequently affords a valuable guide to contiguous vessels. But, while we accept of the assistance that it offers, we can see no utility in preserving it religiously intact, when it is likely to interfere with the free discharge of matter that will probably be formed. We perfectly coincide with Mr. Mayo, in the following opinions and directions.

" In operations for tying arteries, or for the removal of tumours, or in any other case in which the bottom of a wound is not intended or likely to close by adhesion, it is generally preferable to *divide* any portion of muscle which intervenes between the incision of the skin and the bottom of the wound. If the edge of the muscle is *drawn aside* only, in the first place it may still embarrass the operator; in the second place, after the operation, it has a tendency to prevent the free escape of matter from the interior of the wound, and to retard the process of healing. The promptness and completeness with which a divided muscle unites, renders the above rule entirely unobjectionable.

If a muscle has to be divided in an operation in order to reach a part below it, it is better upon the same reasoning to divide it transversely, than to make a longitudinal section, or one parallel to its fibres." 114.

We are sure we have seen mischief from pursuing a contrary plan to that above recommended. The succeeding suggestion, or rather caution, is valuable also.

" In dividing parts in an amputation, to secure a fleshy stump, a certain proportion must be kept between the quantity of integument and muscle left, and the length of bone. But at the time of an amputation, a limb may be in one of two different states; it may be either in full muscular strength, or it may be wasted and extenuated. A different calculation is required for the two cases. For in the first, the muscles retract very little on division; while in the second, they retract to an extraordinary degree. The reverse holds with the integument. In a strong and large limb, over which the integuments are already stretched to the utmost, they fly back to remarkable extent upon division. In an extenuated limb, in which it has already shrunk to the utmost, the skin hardly retracts at all." 114.

Mr. Mayo repeats the recommendation of Mr. Hodgson, of Birmingham, with respect to the management of stumps after amputation. Mr. H. advises the surgeon to leave the cut surfaces of the muscles exposed for twenty minutes, or for half an hour, in order that, before they are brought together, they may be glazed with an exudation of lymph. Union by the first intention is then more likely to ensue. Such protracted exposure, as it lengthens the period of the operation, must of course enhance the sufferings of the patient, and should be practised with discretion by the surgeon. We think it probable that, in many cases, it might do more harm than good, by exhausting and depressing a weakly patient.

*Hypertrophy* of muscles is a rare affection. But Mr. Mayo has seen one instance in which the tongue was affected, and he relates another of a similar description, which occurred to Mr. Hodgson.

*Case.* A child, when about two months of age, was observed to project its tongue beyond the gums, and to sleep with its mouth open. The size of the tongue increased, and the child took the breast with difficulty; but it grew, and appeared healthy. When eighteen months old (July, 1832), the tongue was very large and thick, projecting more than an inch and a half beyond the teeth, and served to widen the mouth considerably. The incisor teeth had formed a deep furrow upon the upper and under surface of the tongue, but ulceration had not taken place. The protruded surface of the tongue, from being constantly exposed, was covered with a coat of dried mucus. There was no appearance of diseased structure in the part. The child was weaned, and fed as other children of the same age (with spoon meat). She was always pleased with animal food, and experienced no difficulty in tearing it to pieces with the upper teeth pressing upon the tongue. There was no pain in the part. In April, 1833, the protruded part of the tongue was as large as a small orange. Mr. Hodgson removed it by the ligature, and the case did well. In May, 1835, the child had learned to read, and could articulate very well.

*Atrophy* of the voluntary muscles presents itself, according to our author, under several forms.

The first is simple atrophy, in which the muscles become small, shrunken, flaccid, pale, and devoid of irritability. Such is the condition of the muscles of wasted or of palsied limbs.

The next form described by Mr. Mayo receives from him the name of "rigid atrophy." In this case, the proper muscular structure wastes, and the tissue which remains is tense, and hard, and inextensible. The common seat of this affection is the sterno-cleido-mastoid muscle; the distortion which it produces is termed the wry neck. But there may be other causes of the wry neck. Other muscles, besides the sterno-mastoid, may be concerned, or the affection of that muscle may be spasmodic. We feel doubtful of the propriety of applying the term "atrophy" to an alteration, in which wasting is of secondary consequence, and perhaps even consecutive in date. The induration of the muscle is the most important and most prominent phenomenon. Hypertrophy and atrophy are terms which should be limited to augmentations and diminutions of the volume of parts, independently of other alterations of moment. Unless those terms are so restricted, the laxity and incorrectness of nomenclature that must ensue is obvious.

We have seen wry neck produced by rigidity and induration of the sterno-mastoid muscle, but we have not enjoyed an opportunity of ascertaining the precise alteration of its tissue after death. We could have wished that Mr. Mayo had related some case in which such inspection was obtained. From the symptoms during life, we were led to suppose that chronic inflammation was present, and the remedies that effected most benefit were those adapted for its removal. We remember one case in which repeated leechings, followed by blisters and by mercurial ointment, were of very great service—indeed, we believe, effected a cure.

Dr. Mott informed Mr. Mayo that he had seen three cases of rigid atrophy in the muscles which raise the lower jaw: the effect of the change of structure had been gradually to fix the mouth in the closed position. He had succeeded, however, by means of a suitable instrument, in forcing the jaws gradually to unclose.

The third and last form of atrophy mentioned by our author deserves, we think, some notice. We will subjoin the observations and the cases of Mr. Mayo, prior to offering any remarks of our own.

"There is another form of atrophy, in which a rapid and total absorption of the muscular structure takes place. The attack has an acute character, and is attended with great pain, which looks like inflammation: yet it certainly is not inflammation of the muscle, though it possibly may be accompanied by, or even result from, inflammation of the membranes of the muscular nerve. The disease is brought on by long exposure to cold. I have witnessed the two following cases.

Thomas Styles, aged twenty-seven, a carver and gilder, four years ago, when heated with exercise, and in perspiration, was obliged in his business to keep the right hand in water for an hour; to which he attributed what shortly followed. He was attacked with pains in the arm, which were considered rheumatic; and the arm was rubbed with stimulating applications, and kept warm in flannel. Weakness of the arm, however, and wasting ensued. The arm and hand gradually became tremulous, and have continued so. He cannot raise his hand to his head, nor extend the arm horizontally, nor supinate the wrist: he has, however, the use of his hand, and can write. The muscles of the arm and forearm generally are extenuated; but the latissimus dorsi and serratus magnus are completely gone.

John Jeremy, aged forty-five, a farmer's workman, accustomed to frequent exposure to wet and to let his clothes dry upon him, four months ago was seized with pain in the left shoulder, which lasted, almost without intermission, for six weeks; it was most severe a fortnight after its commencement: it was at times as violent, he said, as if his arm was coming off. There was no swelling or redness, no numbness, no general tenderness on pressing the shoulder, but great pain was felt on lifting the arm by means of the other hand. The arm he could not lift by its own efforts. About a week from the beginning of the illness, he observed that the shoulder had lessened: the extenuation continued progressive after the pain had ceased; and now the deltoid, supra and infra spinati, and the teretes, appear to have been wholly absorbed, or reduced to thin layers of membrane. The shoulder is free from pain, the joint undiseased, but he has no power to lift the arm: the forearm and hand are not wasted. Pressure on that part of the deltoid which covers the circumflex nerve is now attended with pain; and at the height of the attack, when the suffering was the greatest, used to aggravate it." 118.

We are not aware of the reasons which induce Mr. Mayo to assert, that the affection in these cases "certainly is not inflammation of the muscle." In the instances which he relates, he saw the disease at a late period—four years after the commencement in the first—at the expiration of four months in the second. The causes of the affection—exposure to the influence of wet and cold—are surely not unlikely to produce inflammation of a rheumatic character in the cellular and aponeurotic structures. Nor do we see any thing in the progress of the case, to forbid the supposition that such inflammatory action occurred. Violent pain, increased by motion, and terminating in total loss of power, as well as wasting of the limb, are not improbable concomitants and sequelæ of rheumatic inflammation.

However, our suspicion of the actual occurrence of inflammation in these cases, at their origin, is founded on something more decisive than the foregoing conjectures. The deltoid muscle is frequently the seat of symptoms and of changes corresponding to those described by Mr. Mayo. We have seen at least five or six examples of this disease.

It takes place, so far as we have seen, in persons exposed to wet and cold. It commences, sometimes suddenly and sometimes gradually, with pain in the shoulder, aggravated by motion, and commonly by pressure, and preventing more or less completely the action of the muscle. The symptoms continue with little alteration, and the muscle wastes. In some instances, thickening is felt at its insertion or at its origin—in other cases this is less distinct. The neighbouring muscles are very frequently implicated, and probably the tendons of the scapular muscles, at their insertion into the head of the humerus, as well as the contiguous bursæ, are sooner or later in some degree affected.

We have at this moment under our care a gentleman who labours under this affection. He is an officer in the artillery, and was exposed to atmospheric vicissitudes while on service with his regiment in Ireland. The complaint began with slight pains in the left shoulder. These continued and he found increasing inability to raise the arm. Stimulating liniments, friction, and vapour-baths were employed, but these means only aggravated the complaint. It was some months after its commencement, when this gentleman came under our care. There was then slight thickening and induration at the point of insertion of the deltoid, as well as at the end of the acromion. In both these situations there was tenderness on pressure. There was also tenderness at the origin of the coraco-brachialis muscle. The arm was wasted—the deltoid extremely so. This muscle was absolutely powerless, and none of the motions of the limb that depend upon it could be in any degree performed. We prescribed repeated blistering, with calomel and opium, and other remedies of less importance. At the end of a month, almost all pain and tenderness had disappeared, but still some remained at the origin of the coraco-brachialis. A slight increase of motion was obtained, but the quantity was very trivial. So soon as *all* pain has disappeared, we intend to employ local vapour-baths. We attempted to use the moxa, but the patient had such an insuperable objection to it, that we could not persevere with it.

On the whole, we are of opinion that these affections of the muscles are inflammatory, or at all events, that they often are so, especially in their commencement. The treatment we have found most useful has been local depletion, succeeded by counter-irritation; and, so soon as *all* pain has disappeared, the stimulus of vapour-baths, electricity, and remedies of that description. Yet we must admit that permanent paralysis is greatly to be dreaded, if the malady has made progress before it comes under the surgeon's care.

*Steatosis*, or degeneration of the muscles into fat, is not a very unfrequent occurrence. Sometimes the change is accompanied with such pain, that amputation of the limb has been performed. Dr. Craigie mentions that Vicq d'Azyr found the psoas, iliacus, and other muscles, converted into fat, which is described as white, firm, and contained in numerous minute cells; the uniting cellular tissue, whitish, looser, and more separable than usual: the fat is not deposited between its filaments, but forms part of their substance. Examined by a good glass, it presents a mass of soft transparent fibres of various diameters in different parts of their length.

We need not say any thing on ossification or inflammation of the muscles. Mr. Mayo's remarks are too brief to require notice. But we may extract the following observations on the *entozoa* found in the voluntary muscles.

"The *cysticercus cellulosæ* is found in the voluntary muscles.

The *trichina spiralis*, recently described by Mr. Owen, is an entozoon which appears to infest the voluntary muscles exclusively. The flesh of bodies beset by trichinæ spirales appears studded with minute whitish specks, which are of an elliptical figure, one-fiftieth of an inch by one hundredth, and contain a minute coiled-up worm. A few of the cysts contain two worms. The cysts are supposed to be cellular membrane condensed around the entozoon. The worm is disposed in two to two and a half spiral coils. When straightened, it measures from one-twenty-fifth to one-thirtieth of an inch in length, and from one-seven-hundredth to one-eight-hundreth of an inch in diameter. It is round and filiform, terminating obtusely at both extremities, which are of unequal sizes, and tapering towards one end for about a fifth part of its length. At the larger extremity, Mr. Owen observed a large, transverse, linear orifice or mouth.

The two patients, in the bodies of which these entozoa were identified, died in St. Bartholomew's Hospital, 'after long and debilitating illness, producing great emaciation; unaccompanied, however, with any eruption on the skin, or any greater loss of muscular power than would probably have arisen from the diseases of which they died.'" 119.

The only affection of the *tendons* which requires specific notice is displayed in the following case. A gentleman, thirty-two years of age, after taking more exercise than usual, observed a swelling of the size of half a bean on the inner and anterior surface of the tendo Achillis, which occasionally caused pain in walking. Half a year afterwards, a similar swelling formed in the other tendo Achillis at the same place: the tumors were situated about two inches above the os calcis. He applied a solution of the oxymuriate of mercury in alcohol, which produced soreness of the skin, without making at the time any sensible impression upon the swellings; and then he thought no more about them. They still continue, but are something less in size; and although sensible, so as to be rather painful if pressed, they are otherwise without sensation.

The next chapter is devoted to affections of the cellular tissue, fasciæ, and adipose tissue. The alterations of the former are treated of by Mr. Mayo, under the heads of emphysema—serous infiltration—chronic abscesses—reparation—morbid growths—encysted tumors—and, finally, fibro-albuminous tumors.

The remarks on emphysema contain no novelty. But we will pause at the serous infiltrations which invade the cellular tissue. Of ordinary anasarca we should indeed say nothing, had not Mr. Mayo laid down a principle, which, being contained in an elementary work, requires critical notice. Mr. M. observes:—"Under the operation of various causes, of which the commonest is delay of the circulation from disease of the valves of the heart, the quantity of serous fluid in the cellular tissue may be considerably increased." We are inclined to do more than doubt the correctness of this remark. For one case of anasarca from valvular diseases of the heart, we have seen very many from hypertrophy or dilatation, or both, of that organ. The fact is, that *all* diseases of the heart are apt to occasion anasarca, and as valvular disease is not near so frequent as hypertrophy and dilatation, the latter are of course more ordinary causes of anasarca than the former. Such, at all events, is the conclusion we would form from the facts that we have witnessed.

But anasarca is not the most serious form in itself of serous infiltration into the cellular tissue. Mr. Mayo proceeds to state:—

"There are occasions on which the fluid with which the cellular tissue is infiltrated is not simple serum, but has an acrid and stimulating quality. This is observed in the fluids of the cellular membrane adjoining a part bitten by a venomous serpent; or in the swelling which, in unwholesome constitutions, follows a severe injury, such as a compound fracture; or in the phlegmonous erysipelas.

There is nothing more formidable in surgery than the second case to which I have adverted. An instance is now before me. A labourer, of a full, gross habit, yesterday morning early, was thrown down by a waggon, the wheel of which passed over his knee, causing a compound fracture of the inner condyle of the femur, and opening the knee-joint. He refused to have the limb amputated. At this time, thirty hours after the accident, the thigh, nearly to the hip, is swollen to double its natural size, the veins full, and the skin mottled with large patches of red: the ordinary hue of the skin which attends this form of swelling, when its progress is something less rapid, is a pale coppery tint."

"4. The cellular tissue in phlegmonous erysipelas constitutes a medium along which an acrid humour rapidly finds its way, the presence of which contributes to excite suppuration and sloughing. The cellular membrane is no less the ordinary seat of common phlegmon. Phlegmonous or circumscribed inflammations often occur immediately below the integuments of the arm and of the thigh. The same often form in the nates, and about the anus. A common boil is a small phlegmon, with a central slough of cellular membrane,—a carbuncle, a large and virulent boil." 123.

We are not quite assured of the correctness of these views of diffuse inflammation of the cellular tissue, and of what is denominated "phlegmonous erysipelas." Mr. Mayo asserts that in these affections there is an

"acrid humour" distilled into the cellular tissue, and he implies that the mischiefs that ensue are produced by the acrid qualities of that humour. But of this we see no sufficient proof, and there are many reasons for arriving at an opposite conclusion.

If an individual receives a bite from a rattle-snake, or is inoculated by any morbid poison, and if rapid and destructive infiltration of the cellular tissue follows, it is reasonable to suppose that the fluids of the part have been corrupted, and that mortification of the skin and so forth, are the consequences of their depravation. But if, on the other hand, a man apparently in health, receives a compound fracture, and in twenty-four hours or less, serous infiltration has spread through the limb, the skin of which is mottled and subsequently dies, we do not conceive that we have any right to assume the presence of an acrid fluid. What has so suddenly vitiated the serum? Why should the fluids, which up to the moment when the injury was inflicted presented no sign of acrimony or of alteration, be invested on the instant, and after the occurrence merely of a wound, with such poisonous properties as have been assigned to it? We see no good reason for entertaining this hypothesis, if a simpler one will equally explain the facts.

And a simpler one will, we conceive, explain them. The cellular tissue, from its structure and constitution, favours the rapid diffusion of fluids thrown into it. We are all practically acquainted with the fact, that in strong and healthy persons inflammation of a part is disposed to be arrested by the effusion of coagulated lymph. Setting aside theoretical considerations, this is an admitted fact. If, then, an injury occurs to a person of a bad habit of body, in whom the inflammation is not readily bounded by the deposition of lymph, and if in such a person the injury has affected the cellular tissue, observe the consequences that ensue. An inflammatory serous effusion is rapidly poured into the tissue—the structure of the latter promotes its transmission from cell to cell, and no sufficient barrier of lymph is found to confine it—the cells of the cellular tissue distended with the effusion, and deprived of a proper supply of blood, lose their vitality and slough—the skin, deprived of its vascular support, sloughs too, but *after* the cellular tissue—and, finally, the other tissues are secondarily involved to a greater or a less degree by the extension of the rapid yet low inflammatory process. Such is the more simple explanation we would offer of the phenomena presented by diffuse inflammation of the cellular tissue. To say there is an acrimonious humour generated is to assert what has not yet been proved, and what is in itself exceedingly improbable. The two essential causes of the wide and the rapid extension of inflammatory effusion and its consequences, are, the nature of the cellular tissue on the one hand, and impaired constitutional powers on the other.

5. After observing that chronic abscesses form in the subcutaneous, inter-muscular, and inter-fascicular cellular tissue, and after noticing, in illustration, abscesses in the thigh, in the loins, and psoas abscess, Mr. Mayo proceeds:—

"Another kind of chronic abscess occurs in the cellular membrane. The two following cases may serve to exemplify this disorder. In the first of the two, the inguinal lymphatic glands participated in the disease.

A middle-aged man had for several months a tumour in the groin, which had formed very slowly: it was about the size of two half oranges, but presented three or four rounded elevations : he had experienced little pain in it. Shortly before his admission into the Middlesex Hospital, the skin had become red and softer at one part of the tumour. The swelling had greatly the appearance of malignant disease. The soft and red integument now threatened to slough : I punctured it, when a little air and fœtid ichor escaped. The integument, by sloughing and ulceration, in a few days gaped for some extent, and nodular granulating masses were seen within. No further unpleasant feature, however, presented itself; and without any great quantity of discharge taking place, the swelling slowly subsided, and the part healed.

There is a middle-aged man now in the Middlesex Hospital, who was admitted eight weeks ago, with the calf of the leg immensely swollen, the skin of a dark red, with a small hole in the middle, through which some thin pus found vent. Some months before, he had been kicked by a horse on the calf of the leg, to which he attributed the swelling. Placed in bed, and the leg poulticed, the swelling diminished at first rapidly; the discharge, however, was very inconsiderable in quantity. A probe being introduced passed below the gastrocnemii : the external wound was enlarged a little; the abscess got well. But at the same time this patient showed me a swelling on the back of the fore-arm, as large as, and much of the figure of, two half walnuts : it had been a year forming, and latterly had become painful : it adhered to the periosteum covering the posterior edge of the ulna, and to the aponeurosis. My attention was now drawn to the general health of the patient, and I found that he had had for many months a scaly eruption upon the skin ; and he said that a year before, a tumour like the present, only less, had formed at the corresponding part of the other arm. I gave him, upon this, the decoction of sarsaparilla, with the oxymuriate of mercury : his health began to improve, and the scaly eruption to die away ; but the lump below the elbow increased in size. I therefore punctured it with a lancet in three places, where it was most elastic ; it bled freely, and at one part small fragments of lymph escaped with the blood. The swelling was poulticed, and is now beginning to suppurate, and lessens daily." 125.

We have seen several cases resembling those described by Mr. Mayo. As we consider that description imperfect, we will take the liberty of adding to it what we have had an opportunity of observing.

In persons much deranged in health, and particularly in those who have lived freely, and, perhaps have been rather suddenly reduced by medicine or in diet, it is not very unusual for a diffused swelling to occur in some portion of a limb, most commonly in the calf of the leg. There is obscure fluctuation, sometimes scarcely exceeding the *bogginess* noticed in diffuse subcutaneous inflammation—the skin is of a darkish red colour, without any defined erysipelatous margin—there are excessive pain and tenderness on pressure—and the general symptoms are of a low, if not actually a typhoid character. In the calf of the leg, in which we have seen this form of abscess, it was seated between the gastrocnemii and the deep flexor muscles. The matter was dark, and the cellular tissue sloughy. In one patient there was obstruction in the femoral artery, in consequence apparently of calcareous deposition in its coats, and coagulation of the blood within it. One patient with this form of abscess died and enabled us to ascertain that there was only a sort of sloughy abscess in the situation we have mentioned; the other patients that we have had an opportunity of seeing recovered.

The preceding is one form of abscess occurring in the cellular tissue in persons in a cachectic condition. We will mention a case that displays another description of abscess.

An old man was received into St. George's Hospital with what seemed to be a tumor on the side of the neck. It was of considerable dimensions, lobulated on the surface, and presenting in several of the lobular masses a feeling exactly resembling that communicated to the touch by fungus hæmatodes. It was thought to be a fungoid tumor. After a short time a slight erythematous blush appeared upon one of the most prominent lobules; the skin ulcerated; a whitish mass was discharged, evidently lymph entangled in dead cellular tissue; suppuration and granulation followed; and the cavity gradually filled up. The same thing occurred with the other lobules in succession, in some there being lymph and pus, in others merely lymph. Thus the disease was cured. The patient was in a very cachectic state, and the general treatment consisted in the exhibition of stimulants and tonics.

A species of cachectic abscess similar in some degree to the preceding, has sometimes been called the "subcutaneous tubercle." The patient is almost always in bad health, and frequently in the state resulting from abuse of mercury. In different parts of the body, but more especially in the lower extremities, rounded nodules are felt immediately beneath the skin, to which they communicate a more or less distinct projection. These nodules vary in size from that of a large pea to that of a good-sized walnut. At first they are scarcely tender on pressure, and the skin is not discoloured, but as they increase a little redness appears upon the skin, which gradually grows thinner, and at length ulcerates. The aperture thus formed is not large, and displays a whitish deposition in the subcutaneous cellular tissue. This is unorganized lymph, opaque, and sometimes of caseous consistence. The cells in which it is deposited die, apparently from their vascular supply being cut off. After the ulcerated opening has attained sufficient size, the lymph and slough come out, and a granulating cavity follows. This is the course which the majority of the tubercles run. Some, however, even after attaining a certain magnitude, become absorbed.

We have mentioned these facts because Mr. Mayo has only imperfectly described the varieties of suppuration to which the cellular tissue is subject. In fact, we may observe that the diseases of this tissue have not hitherto been well described, numerous as the descriptions of them have been.

Passing over a notice of the reparation of cellular tissue, and of encysted tumors, we may pause at Mr. Mayo's description of a fibro-albuminous tumor which occurs in it.

"Another growth, and which is perhaps confined to the cellular tissue, may be termed fibro-albuminous tumour. It sometimes occurs in the cellular tissue of the breast, and grows to a considerable size, having much of the external character of malignant disease. In the following instance, this growth occurred in the cellular tissue of the parietes of the abdomen.

William Latham, ætat. forty-seven, was admitted into the Middlesex Hospital, in July 1835, with a tumour upon the umbilical and left iliac region. It is twenty years since he first noticed a small lump below the skin, of the size of a pea, which gradually enlarged, but was not painful. Six months ago, it was as large as a small egg; and a surgeon, whom he consulted, tried to break it by pressure. The skin then gave way, when the swelling enlarged rapidly; at the time of his admission, the swelling looked a great fungoid tumour, five inches in diameter, an inch and a half in thickness, and adhering by an oval pedicle three inches in its long diameter. The surface of the tumour was lobulated, and covered with large flat granulations, of a pale red colour, which secreted matter. The tumour had bled frequently and profusely, not from the whole surface, but from two or three points. Since it had been broken, the patient had frequently experienced shooting pains in it, which lasted two or three minutes. He was greatly reduced by the bleedings and the discharge. The skin round the pedicle of the tumour was mottled of a dark red, and several tortuous and swollen veins extended from it in different directions. The inguinal glands were not enlarged.

I removed this tumour on the 23d of July. It was situated upon the external oblique muscle and its tendon, from which I dissected it: two vessels only required to be tied. The wound granulated healthily; and a few days made a remarkable improvement in the patient's appearance and strength, who is now [August 10] all but recovered.

On examining the tumour, it was found to consist of two distinct masses; one of the size of a walnut, seemingly of later formation, contained in a clean membranous capsule, firm, of no great vascularity, grey, semi-transparent, elastic, easily breaking down with pressure. The other, which had formed the more prominent and larger mass, was of much greater size: its base was covered by integument, the giving way of which, six months before, had allowed of or given rise to its rapid increase, and exposed it as a vascular and secreting surface. In one part of the larger mass, and inclosed in it, there was a second growth, opaquer in colour, and more fibrous in character, not unlike, though less firm than, the ordinary fibro-cartilaginous tumour of the uterus.

I assisted Mr. Broughton in removing a large tumour from the thigh of a female, which had very much the character of the smaller and more fibrous portion of the tumour described above. But it was of a more gristly texture, or more closely approximated to the fleshy tumour of the uterus. It appeared to have formed in the intermuscular cellular tissue." 127.

Experience, we fear, does not materially increase our confidence in the artificial distinctions which are drawn, between the various forms of sarcomatous tumor. The pathologist traces minute discrepancies between tuberculated, pancreatic, scirrhous, and medullary deposites, but their diagnosis previous to their removal is uncertain, and the final result of operations is precarious. When some marked distinction between the mass we have extirpated and true malignant formation exists, we too often find that malignant growths follow in other organs notwithstanding; and occasionally, though unfortunately, very rarely, what appear to be genuine cancerous tumors are extirpated with permanent success. In the midst of this uncertainty, the surgeon is compelled to look upon all these tumors with suspicion, and to distrust those flattering promises of cure, which reliance on our powers of discriminating the malignant from the non-malignant growths is calculated to engender.

The affections of the aponeuroses and fasciæ are summarily disposed of by our author. Yet not so summarily but that criticism may take exception at the doctrines.

Aponeuroses, observes Mr. Mayo, are liable to simple and specific inflammation. "The first is the most frequent untoward consequence of ordinary bleeding. The wound festers, the arm and fore-arm adjacent to the elbow are swollen and painful, and the skin has a slight blush on it." This is the echo of the common notion; yet we doubt if that notion is correct. Aponeuroses and fasciæ are certainly not *liable* to inflammation, or rather they are not prone to it. After injuries of a limb, it is the cellular tissue that commonly inflames, and the fasciæ suffer in consequence. If such is the case after injuries in general, why should we suppose that the prick of a lancet exerts such a peculiar influence on the fascia of the arm? Is there any thing in that weapon that should render it so obnoxious to the fascia, or is there any thing in that fascia which should make it more susceptible of inflammatory action than the fasciæ are elsewhere? If there be, we do not know it. But we have better reasons for believing that it is *not* the fascia which constitutes the essential seat of the inflammation, than reasoning of this description. We have examined arms after death in which such symptoms as Mr. Mayo has alluded to occurred. The inflammation and the suppuration were in the cellular tissue, above the fascia or below it. If above it, the fascia was little affected; if below it, just as much as it usually is in subfascial inflammation of other parts. The fact is that Mr. Abernethy's descriptions of inflammation of the fascia are hardly consistent with the present state of pathological knowledge.

The only observations on the alterations of the adipose tissue, requiring any notice, are comprised in the following case, communicated to Mr. Mayo by Mr. Samwell. The case in question exhibits a peculiar and rare deposition in the adipose tissue.

"A female, ætat. thirty-six, after suffering from rheumatism, had a swelling of one instep, which, however, went away; but shortly after, the leg began to enlarge, and became painful. The increase in size was very gradual; but in four years the leg had become twice as large as natural. The swelling was diffused over the whole leg, but was greatest upon its outer aspect: to the touch it felt elastic, like Indian rubber. The skin was not discoloured. Mr. Samwell punctured the swelling, when a thick, semifluid substance, like grumous blood, escaped; upon which he enlarged the puncture to a long incision, and squeezed out more of a similar nature. The wound healed readily. But the swelling, at the side where it had been opened, filled again, and the skin began to ulcerate. The limb was then amputated above the knee; the patient recovered, and has been four years well.

Upon examining the swelling, it was found to be exterior to the fascia, and situated in the adipose tissue; which, where the enlargement was greatest, was three inches in thickness, soft, grumous, and red. At the extremities of the swelling, the adipose tissue seemed infiltrated with a gelatinous substance only. Intermediately, there was a transition in colour between the pale gelatinous swelling of the adipose tissue near the knee and ankle, and the sanguinolent and grumous effusion in the middle of the tumour. No other tissue appeared to be diseased." 129.

From the adipose tissue, Mr. Mayo passes to the nervous. To that he dedicates one hundred pages, and concludes the present Part with the pathological alterations of the skin. The latter is more naturally considered in connexion with the cellular tissue and the adipose, and we shall pass over the morbid anatomy of the brain and nerves, for the purpose of uniting the textures that Mr. Mayo has severed, perhaps with unnecessary violence. As the text of Mr. Mayo continually presents us with opportunities of introducing observations that illustrate, or that oppose it, we shall not have room in the present article, for the consideration of the organic alterations of the nervous system. We shall defer our notice of them to the ensuing number of this Journal.

Our author remarks, in reference to the alterations of the skin, that they can be scarcely deemed adapted for enumeration and description in an elementary treatise. Many are too trivial, and many too important, for the crippled notice that such a treatise admits of. He divides the subject into three sections—affections of the skin which have points in common with the pathology of other organs—the eruptions of the skin—and, finally, its principal ulcers. Almost all the second and a part of the first section are avowedly built upon Rayer's work. Our notice of this chapter will, therefore, be brief and unconnected.

In the observations on injuries and reparation of the skin, we observe the following passage. After alluding to the condition familiarly known as chilblain, Mr. Mayo remarks :—

"White gangrene is an affection of the skin, in which, without any assignable csuse or preliminary symptom, patches of skin, of the area of one, two, or three square inches, suddenly die. White gangrene occurs sometimes on the breast. In the museum of King's College there is a model of white gangrene, in which the disease attacked the arm, several patches of cutaneous gangrene successively forming: the sphacelated parts were white from the commencement of the process to their separation, when healing and healthily granulating sores were left." 231.

Probably the great bulk of our readers would scarcely comprehend the nature of the affection to which the preceding passage alludes. We must regret that that passage is so brief, referring, as it does, to a disease which, so far as we know, has not yet been sufficiently investigated or clearly described. We have seen two instances of this curious malady, and, as we possess the notes of one, we will take the liberty of introducing here a succinct account of it.

*Case.* A girl, aged 18, was admitted into St. George's Hospital in Sept. 1830, under the following circumstances. For a month prior to her entrance into the Institution, she had several attacks of gangrene of the skin of both arms. The gangrene made its appearance in the following way :—

First, a diffused redness, with rather a glossy appearance on the surface, shewed itself on some part of the skin. This was sometimes preceded by shivering and fever. On the second day, patches of a pearly or blueish-white colour made their appearance in the centre of the redness; they became confluent, and so a large whitish patch, three or four inches in length, and about two in breadth, with a red margin around it, was produced. There was no elevation of the cuticle. In six or seven days, the white patch of skin was surrounded by a groove, formed by ulcerative absorption. Afterwards the inclosed cutis was thrown off as a slough, leaving a clean granulating surface, to all appearance the deep layer of the cutis.

If left without covering, the pearly white patches became quite dry, and looked like parchment. Some seemed to have a slight layer of pus beneath them—others to have a serous fluid as their substratum. But it was dubious whether either was really present.

The health of the patient was good, excepting when she suffered from the occasional pyrexia. Her complexion was rather fair, and inclined, perhaps, to strumous. The menstruation was regular. She had lived for two years in London. She had not been confined to any particular description of diet. She had taken for the affection opening medicine, aloes, and bark.

On the 12th of October, a fresh invasion of the disease occurred, and the following phenomena were exhibited. On the dorsum of the right foot was a patchy, erythematous redness, extending a little way up the front of the leg. In the centre of this were here and there the dead-white patches, looking as if a solution of chalk had been dropped upon the part and dried there. The surface of these patches had a slightly scaly appearance. There was much tenderness on pressure, and some swelling of the dorsum of the foot, from slight subcutaneous effusion. The pulse was full.

On the 13th, the white patches had extended somewhat, and assumed more of the appearance of minute scales. The redness around had become more circumscribed, and displayed here and there minute ecchymoses. There was an indistinct sensation communicated to the finger of fluid being effused beneath one of the white patches; but, on puncturing it with an exploring needle, this was ascertained not to be the case.

On the 14th, the surrounding redness had subsided into a kind of *cordon* of ecchymosis, immediately encircling the white patches. This ecchymosed part was sensibly elevated above the level of the latter.

On the 16th, two or three litttle islands of cutis, sound, but exhibiting ecchymosis, had appeared in the centre of the white patches. In these spots, the cutis had evidently not quite died, and was recovering itself. On the 19th, the ulcerated groove at the margin of the white patches had made its appearance.

In the night of the 23d she had a rigor, succeeded by pyrexia. A similar occurrence in the night of the 26th was immediately followed by erysipelas of the leg and foot. The erysipelas did not entirely disappear until the 18th of November. The sloughs of skin had, in the interim, been thrown off, and the ulcerations had healed. The leg was swollen. On the 22d she was made an out-patient, and we have not, since that period, heard of her.

We have given this case at some little length, because it exhibits tolerably well the characters of a peculiar gangrene of the skin, which has probably not presented itself to the notice of many of our readers. In another case which we witnessed, precisely similar phenomena were displayed. The gangrene of the cutis was immediately preceded by an erythematous blush. In neither instance did the whole substance of the cutis seem to suffer, the slough appearing to be limited to its superficies, and granulations afterwards springing from its chorion.

The causes of the complaint are involved in obscurity. In neither of the cases which came under our observation, was there any thing appreciably wrong in the system or the part. In the girl whose case we have related, menstruation was regular; in the other patient (a female of about thirty years of age) the catamenia were deficient in quantity, and did not appear so often as they should do. The remedies tried in the hospital were bark, and various emmenagogues. They appeared to exercise very little influence over the disease.

*Hypertrophy of the Skin.* Mr. Mayo refers to the skin of a person whose arm is modelled in the King's College museum. It is thickened upon the back part of the limb, of a brown colour, and hangs in a thick pendulous flap several inches in length. The hypertrophy was congenital.

The integuments of the nose are particularly liable to enlarge and thicken. After the removal of a part of the hypertrophied mass, the wound cicatrizes wholesomely. Mr. Mayo remarks that, in the elephantiasis of the Arabs and in the Barbadoes leg, the skin is the seat of the hypertrophy. In two dissections of the Barbadoes leg, which we made, the principal growth was in the subcutaneous adipose tissue.

Hypertrophy of the papillæ of the skin occasionally succeeds or accompanies ulcers of the legs, the application of blisters, eczema, and impetigo. The papillæ become particularly apparent when the part affected is plunged in water, and look mamillated and uneven, like the pile of coarse plush or velvet. The skin in these cases is habitually covered with scales of epidermis, sometimes micaceous in appearance, generally brown, and easily rubbed off.

Unnatural blackness of the skin has been met with. Mr. Mayo quotes the case.

" A lady, says Lecat, about thirty years of age, became pregnant. About the seventh month the forehead assumed a dusky hue, of the colour of iron rust: by degrees the whole face became entirely black, except the eyes and the edges of the lips, which retained their natural colour. The hue was deeper on some days than others. This lady being naturally of a very fair complexion, had the appearance of an alabaster figure with a black marble head. Her hair was naturally exceedingly dark ; but the part of it which grew from the dark-coloured skin appeared coarser, and filled with a blacker sap than the rest, to the height of about a line or two above its roots. She did not suffer from headach ; the appetite was good ; the face after becoming black was very tender to the touch : the black colour disappeared two days after her accouchement, with a profuse perspiration, by which the sheets were stained black. The child was of a natural colour. In the following pregnancy, and even in a third, the same phenomenon re-appeared in the course of the seventh month : in the eighth month it disappeared ; but during this month the lady became subject to convulsions, of which she had an attack each day." 233.

The skin has also assumed an unnatural whiteness, both in blacks and in Europeans. The precise causes of these changes of colour do not appear to be determined. The slate colour produced by nitrate of silver seems to be seated in the chorion. Mr. Brande detected oxyde of silver in the stained tissue.

Scirrhus, medullary sarcoma, melanosis, occur in the skin. This is the seat of what is denominated cheloïd tumor.

" A reddish point, of the form and dimensions of a grain of barley, rising on some point of the healthy skin, or on the cicatrix of a small-pox pustule or burn, is first observed, which slowly enlarges, its surface covered with numerous transverse wrinkles, but smooth. Increasing, it preserves its redness and hardness, and shapes itself either as a broad cylindrical crest or ridge, or as a flattened tumour, sending off prolongations from its circumference, having some resemblance to the claws of a crab. This growth is of rare occurrence; it does not ulcerate; when removed, it has returned : left to itself, it has after some time been found to shrink." 236.

The perspiration varies in its sensible qualities. All are familiar with the excessively acid perspiration of rheumatic fever—with the very offensive perspiration of ague. In persons labouring under the purulent deposites, the perspiration has a sickly odour, of a very peculiar description. But probably few have seen *blue sweats.* Such a case has been related by Ballard, and is transferred to the pages of our author.

A laundress, aged 16, stout in person, pulse regular, catamenia natural, strength and appetite good, but with a loaded tongue and dry cough: presented on the neck, face, and upper part of the chest a beautiful blue tint of the skin, principally spread over the forehead, alæ nasi, and round the mouth. When these parts were wiped with a white towel, the blue colouring matter was detached fron the integument, and stained the towel, leaving the skin white. The blueness of perspiration had commenced two years previously, when she entered on her profession of washerwoman.* After repeated venæsection, sulphur and sarsaparilla were given without benefit. The forehead, face, neck, and belly now became shaded with azure blue, which spread like clouds, and appeared deeper or paler according as the cutaneous circulation was accelerated or retarded; when for example the patient should have blushed, the face became blue instead of red. The face, the fore-part of the trunk, the shoulders, the arms, and a portion of the thighs were alone coloured. The linen of the patient was stained blue. Shortly after this the urine became very scanty, and for three days the patient did not pass a drop; the blue colouring matter became more abundant: there was profuse perspiration at night. This patient experienced great amendment when taking several doses of bicarbonate of soda, which had been found by experiment to neutralize the colouring matter. At length the face only retained a slight blue discoloration, which was increased by exposure to heat, agitation of mind, fatigue, or the catamenial period. The blood that was taken from the arms had the usual appearance. Some that she vomited contained a sufficient quantity of blue colouring matter, to stain the sides of the vessel.

The sebaceous secretion may be morbidly increased—or it may be morbidly altered in quality. In the latter instance, where the face is commonly affected, the skin of the cheeks, nose, or eyebrows, appears covered with a kind of yellowish scurf, nearly of the colour and consistence of the cerumen of the ears. The skin looks thickened and unctuous around this deposit, which in some points is moist and oily, and in others of the consistence of yellow wax.

We pass over the catalogue of eruptions of the skin. We suppose it was necessary to introduce the chart, in order that the pathological cabinet might be complete. But, whether that was required or not, we cannot extend the same indulgence to the attempt to give a sketch of syphilis. The surgeon acquainted with this singular disease will smile, when he is informed that Mr. Mayo writes of primary syphilitic ulcers thus.

"They present four varieties:

*a*. A circular ulcer with raised edges, the base and edges soft, commonly preceded by a pustule.

*b*. A circular ulcer, raised above the level of the skin, without hardness.

*c*. Excavated ulcer: the base and edge hard, as if formed of a cup of thin cartilage; commonly surrounded by a red vascular line; with little secretion. *Hunterian chancre.*

*d*. An ulcer spreading rapidly in circumference and depth, the surface soft and yellow, with red points showing through the secretion, the base and edges

* The Printer's Devil suggests that the complaint might depend on the use of the "blue-bag."

soft: the edge of the skin red and inflamed, and often hard. *Phagedenic ulcer.*" 261.

There is no hesitation, no reserve, in laying down the preceding as *the* syphilitic sores. Yet, if we were asked to point out an arrangement open to objection, we should lay our finger on this. It possesses in almost an equal degree the sins of omission and of commission. Some of the most remarkable syphilitic sores are peremptorily shut out, while varieties differing only in accidental circumstances, are as arbitrarily let in. But we quarrel with the dogmatic tone of Mr. Mayo. He seems to entertain no doubt upon a subject, which perplexes most those who most study it. And his therapeutic precepts are as positive as his diagnostic. There are, he says, a sthenic and asthenic phagedænic ulcer. In the former case, bleeding rarely fails to arrest the progress of the disease—in the latter, cauterization by nitric acid. Mr. Mayo has here generalized absolutely on the observations of others. A more extended experience in syphilis will convince him, that these two turnpike roads are neither so broad nor so straight as he supposes. We will not criticise two cases adduced in illustration—striking examples of the consequences that must result from such notions and such doctrines on phagedæna. In the next edition of these Elements of Pathology, we would recommend our author to expunge the section upon syphilis, with which he is too imperfectly acquainted to allow him to speak with certainty or safety.

Here we must conclude our present notice of the work. If our last words have been those of censure, that was the effect of accident. Its merits as a manual are far from inconsiderable. In our next we shall refer to it again, and present a sketch of the pathology of the nervous system.

---

We now turn to the Fasciculus of the work of Dr. Carswell. It is dedicated to the subject of—

### Hypertrophy.

Hypertrophy may be defined an excess in quantity of the solid materials of some portion of the body; it is opposed to atrophy, which is its converse. Hypertrophy may be simply a matter of physiological consideration, or it may amount to a morbid state, interfering with the functions of the organ it invades, and forming a legitimate object of pathological investigation. It is chiefly in the latter point of view that Dr. Carswell regards it.

Dr. Carswell does well in avoiding a lengthened and unsatisfactory discussion on the *quomodo* of hypertrophy. When we say that it appears to result from an excess of the nutritive function of the part, we advance as far as we can safely go. We know so little of the modes of vital action, that our terms can express nothing save some obvious facts, of the nature of which we are profoundly ignorant. The process by which a part attains its natural form and dimensions, and, when attained, preserves them, we denominate nutrition. How, or where, or why the molecular deposition occurs, is a mystery we cannot solve. Such is the case with hypertrophy. It appears to result from an excessive action of those vessels which naturally nourish the part; and this is all we can venture to affirm.

It appears certain that an increased supply of blood is requisite for the production of hypertrophy. The vessels which proceed to the affected organ commonly augment in volume and capacity. In hypertrophy of the muscular tissue, this is distinctly the fact. Every one who has injected limbs knows how much more readily the wax is forced into the arteries of a muscular subject, than into those of a person in whom the muscles have been little used. Great employment of an organ, particularly of a muscular organ, tends to occasion its hypertrophy. This is seen in the muscles of voluntary as of involuntary motion. The arms of the blacksmith, or the waterman, and the legs of the opera-dancer, shew the influence of exertion on the former—the hypertrophy of the heart in those who have undergone great toil—and the increase of the muscular coat of the bladder in those who have suffered, from any cause, under difficulty or frequency of micturition, exhibit analogous effects in the muscles which are not controlled by the will.

*Origin and Causes of Hypertrophy.*

"Hypertrophy," says Dr. Carswell, "occurs under circumstances so very various and so different in kind, that a correct estimate of its nature, in individual cases, could not be obtained without a previous knowledge of the causes in which it originates. The most remarkable difference in this respect is observed in that form of hypertrophy from arrested or imperfect development, in consequence of which an organ retains after birth that relative condition of bulk which was necessary to its accomplishing a function peculiar to the fœtal state of existence. This is observed sometimes to occur in the heart, an organ which, before birth, is in that state of hypertrophy called concentric. The thickness of the walls compared with the capacity of the ventricles of the heart in the fœtus, is nearly twice that of the walls compared with the capacity of the ventricles of this organ in the adult. This relative disproportion between the walls and cavities of the fœtal heart is conspicuous in infancy and childhood ; but during the regular development of the body, it gradually merges into those proportions of capacity and bulk which are regarded as indicating, in these respects, the normal state of the heart. If, therefore, the changes which should take place in the regular development of the heart after birth are prevented, it is obvious that the permanence of the state of the fœtal heart which we have described, must, under new and so very different conditions of life, constitute a pathological state, a state of concentric hypertrophy, and give rise at an early period to those derangements of function which follow as the consequences of this disease ; and that such is the origin of some cases of this form of hypertrophy is proved by a careful examination of their early history, and of the organ itself after death.

It is worthy of remark, that what may be called *congenital hypertrophy*, may be accompanied either by an increase or a diminution of the bulk of the heart. I have not, however, found that the bulk of this organ, compared with the size of the body in such cases, was very much increased. I have met with it oftener of the normal bulk, and once so small, in the body of a female above the age of forty, that it did not exceed the dimensions which it usually presents at the age of twelve or thirteen."

The causes of hypertrophy may exert a local or a general influence—in other, and more correct phraseology, hypertrophy may affect a part from causes evidently acting exclusively upon it; or it may affect, at the same time, many parts from causes of a less particular description. As an instance of the latter, we observe the enlargement of the bones, lymphatic glands, the upper lip, and other organs in scrofula. The excessive growth of the adipose tissue that occurs in some individuals, must also depend on some general cause.

The local causes of hypertrophy are arranged by Dr. Carswell under three heads—1st, the frequent and increased action of an organ in the normal exercise of its function; 2d, the existence of a mechanical obstacle to the accomplishment of the function of an organ; 3d, the long-continued influence of a morbid stimulus. Hypertrophy under the influence of one or other of these causes occurs in almost all the organs and tissues of the body. It may affect an entire organ, or one or more of its elementary tissues—exist as the only perceptible lesion—or be accompanied with other diseased states.

The examples of hypertrophy from extreme action of the organ in the performance of its natural function, are so numerous and so familiar that they scarcely require enumeration. The legs of the danseuse—the arms of the waterman—the hearts of those who have been subjected to excessive corporeal exertions, all afford instances of hypertrophy of this description. We see the same thing in viscera or in parts which are required to do more than usual work, in consequence of defects in other viscera or other parts. If one lung, for example, be compressed, the other is enlarged—if one kidney is diseased, the other increases in dimensions. We need not multiply instances of this sort.

The second kind of hypertrophy, that resulting from a mechanical obstruction to the accomplishment of the function of an organ, is very common. The heart becomes hypertrophied, from contraction of the aorta, or of the pulmonary artery, or of the auriculo-ventricular apertures. Contraction of the pyloric orifice of the stomach and of various portions of the intestinal canal, from the gradual development of accidental formations within their parietes, or projecting from their internal surface, or from the cicatrization of ulcers, more especially of the latter, are by far the most frequent causes of hypertrophy of the muscular coat of these organs, and which, in consequence of the accumulation of their contents, is always accompanied by an increase of capacity or dilatation. Diseases of the prostate gland and stricture of the urethra, which obstruct for a long time the passage of the urine, and occasion an accumulation of this fluid in the bladder, are followed by hypertrophy and dilatation of this organ to a greater extent than is observed under any other circumstances. Obstruction in the course of the ureters occasions dilatation of the canal above, and thickening of its parietes. A similar enlargement is seen under similar circumstances in other excretory ducts. Dr. Carswell relates a remarkable case of dilatation of the lymphatics in the groin.

"M. Amussat, of Paris, was called to a young man, about twenty-six years of age, who the day before had been seized with severe pain in the abdomen, followed by frequent vomiting. These symptoms, and the presence of two swellings, one in each groin, nearly as large as an orange, left no doubt that the patient was labouring under the effects of strangulated hernia, but the state of prostration was such that reduction by an operation was not attempted. On examining the patient after death, the only remarkable circumstance observed was enormous dilatation of the lymphatics from both groins upwards, including the thoracic duct. The two swellings situated in the groins, and which at an early age of the patient had been treated as a case of *double herniæ*, (for we afterward learned that he had worn a double truss from his boyhood,) were found to be produced by great dilatation of the lymphatics of the inguinal glands.

When cut into, instead of having a compact structure, they presented the appearance of a coarse sponge, from the size of all these vessels being increased, the most of them presenting from one to three lines in diameter. All the lymphatics of the pelvic and lumbar regions presented the same alteration in a still more remarkable degree. None of them were less than two, many of them from three to four lines, and the thoracic duct was from six to eight lines in diameter. As no obstacle was found in the course or at the termination of the thoracic duct to account for the dilatation of the lymphatics in this singular case, and as these vessels had undergone no other perceptible change, I am disposed to consider it as an example of *malformation* of these vessels, more especially when these facts are connected with the circumstance furnished by the history of the case, viz. the existence, at an early period of life, of the enlarged state of the inguinal glands, the nature of which I have explained."

The third variety of hypertrophy from local causes, or that arising from the long-continued influence of a morbid stimulus, is of constant occurrence in the cutaneous, mucous, cellular, fibrous, osseous, and glandular organs—not unfrequent in the involuntary muscles—and is occasionally seen in the brain, the nerves, and their ganglia. Dr. Carswell, however, observes that it is often difficult to discriminate this simply hypertrophic state, from that in which inflammatory action has led to the deposition of coagulable lymph which has subsequently become organized.

Examples of this sort of hypertrophy are common in the cutaneous and mucous tissues, in chronic ulceration of both, and in irritation and chronic inflammation of the latter, especially of the mucous membrane of the air-passages, digestive canal, and urinary bladder. Not only may the whole tissue be rendered hypertrophic, but its component parts may be unnaturally developed, and its texture, thus magnified, may become more apparent and distinct than in its normal state. Andral has related an interesting example of this species of hypertrophy in the skin of the right leg, which, thirteen years before had been ulcerated, and had afterwards greatly increased in size. On examining the thickened skin from within outwards, it was found to consist, 1st, of the *cutis vera*, much thickened ; 2d, of the *papillary tissue*, which, distinguished with difficulty from the former in the natural state, was in many points much enlarged and elongated ; 3d, of three layers situated between the latter and the epidermis, differing from each other in colour, consistence, and structure. The first of these layers covering the papillary tissue was considered to be analogous to that which Dutrochet has called the *epidermic* layer of the papillæ ; over this again was situated another layer of a dark grey or brown colour, having a reticular structure, occupying the place of, and obviously analogous to, the *rete mucosum ;* and lastly, a much thicker and harder layer than the two former, which, existing only in a rudimentary state in man, presents various degrees of development in animals, and contributes to the formation of shell and horn.

A similar development, from chronic inflammation, is observed in the elementary structure of the mucous membrane, as well as of its follicles and villi. In the mucous membrane of the bronchi and of the urinary bladder, in which villi are not distinguished in the state of health, they become developed when hypertrophy takes place.

Hypertrophy of the cellular tissue from irritation, or chronic inflammation, is probably the most common of all. The subcutaneous and submucous cellular tissue are most frequently affected. Serous membranes do not seem to be liable to hypertrophy, nor is it marked in the fibrous membranes or in tendons. Dr. Carswell looks on the atheromatous and steatomatous deposites which occur on the surface of the middle coat of arteries and veins, or between the middle and the inner coat, as examples of hypertrophy of the former. Such at least is the interpretation which we put on his expressions, which are obscure and vague. If we do not misunderstand Dr. Carswell, we must beg to differ from him. Those deposites are as obviously a new formation, as the cheesy matter of scrofula, or tubercle, or any other morbid production. Even granting that the middle coat becomes thickened from the irritation excited by the presence of these new substances, that is clearly the result of chronic inflammation, and the tissue is not only thickened but altered. Now hypertrophy, if it is to be considered a substantive complaint, must consist in an increase of the natural structure of the part. If augmentation of bulk from alterations of structure be looked on as hypertrophy, there can then be no limit to the term, and all morbid growths, all organic changes attended with increase of substance, must necessarily be included in this generic designation. So far as we have seen, the alterations of the middle coat of arteries have not been fair instances of simple hypertrophy. Most frequently, the middle coat is rather absorbed than thickened, but when thickened we have found the structure altered, and the properties which depend on the natural structure, elasticity and contractility, sensibly impaired. Dr. Carswell presents a description of the changes which he has seen in cases of aneurism of the heart, which is not unworthy of attention. The quotation is unavoidably long.

"The first perceptible lesion in aneurism of a portion or of the entire walls of one ventricle of the heart, (of which I have met with several examples, and always in the left ventricle,) is in every respect similar to that which so often precedes the formation of aneurism of the arteries. The serous membrane lining the internal surface of the ventricle, presents within a circumscribed space, varying from a quarter of an inch to one or two inches in breadth,—or, as in two examples which I have met with, from one-half to two-thirds of its entire extent,—a pale-straw colour; it has become opaque, is closely united to the cellular tissue beneath it, which presents the same colour, and is considerably thickened. Occupying the situation in which these changes are perceived, and sometimes nearly to the same extent, are one, two, three or more depressions, cavities, or sacs. These are lined by the serous membrane and cellular tissue, which appear as if they had been gradually extended and pushed outwards, carrying before them and ultimately causing atrophy of the muscular substance of the heart, the only change which it suffers in such cases. The formation of the cavity or aneurismal sac depends obviously on two causes; 1, the loss of resistance in the serous membrane from disease; 2, the muscular force of the heart, under the influence of which and through the medium of the blood the diseased membrane must yield, be forced outwards, and by compression interrupt the capillary circulation in the muscular tissue, which will become atrophied by interstitial absorption, and thus favour the continual operation of the mechanical cause. While this process is going on, the serous membrane and its accompanying condensed cellular tissue are gradually elongated, and form a smooth though sometimes unequal covering to the sac, the fundus of which is sometimes not larger than the opening by which it communicates with the cavity of the ventricle, at other times much larger, in which case only it has been found to contain coagula or a quantity of fibrine.

Besides the morbid conditions of the internal lining membrane and its sub-cellular tissue which give rise to circumscribed aneurism of the heart, there are others, connected however with these, and having apparently the same origin, and which I have always found to accompany the uniform dilatation of the ventricle in the direction of the affected walls. In the cases in which I have observed this form of dilatation, the opposite surfaces of the pericardium were intimately united by firm cellular tissue, the consequence of a previous attack of pericarditis. The lining membrane of the heart and its sub-cellular tissue presented the same appearances as in circumscribed aneurism; but there was, besides, a greater or less quantity of cellulo-fibrous tissue, occupying the situation of the muscular substance of the ventricle, sometimes throughout the whole thickness of its walls. It is in the situation of this tissue, as I have said, that the dilatation takes place. That this as well as the circumscribed form of dilatation originates in inflammation, appears evident from the circumstances already stated, but more especially from the fact just noticed, viz. adhesions being always found to accompany the latter form. It is, however, important to remark, that the formation of the cellulo-fibrous tissue which takes the place of the muscular tissue of the heart, does not originate in the effusion and organization of coagulable lymph. I have been able distinctly to trace it to the separation of fibrine from blood forced into the substance of the heart, in consequence of softening and rupture of some of its muscular fibres. The gradual transformation of the fibrine into the cellulo-fibrous tissue was very conspicuous."

We are disposed to question the accuracy of Dr. Carswell's position—that dilatation of the heart, whether circumscribed or diffused, originates in chronic inflammation. Whatever Dr. Carswell's opportunities of observation may have been, this must from the nature of the case be an assumption. If Dr. Carswell could prove that partial dilatation of the parietes of the heart is always preceded by a morbid state of the lining membrane, he would have to shew that this is necessarily inflammatory. But he cannot prove the former postulate: his experience will not sanction the attempt. We remember examining the body of a gentleman who died from rupture of an aneurismal pouch of the left ventricle. The hæmorrhage took place into the pericardium. In that instance we could detect no morbid change in the lining membrane of the ventricle. The muscular substance appeared to be absorbed, the walls of the pouch were extremely thin, and under the influence of exertion they had given way. First then, Dr. Carswell cannot possibly assert that all cases of aneurism of the heart result from alterations of its lining membrane; and, secondly, even if he could do that he must still shew that those alterations arise from inflammatory action. This is probably the case when we discover the traces of previous pericarditis, but when, as not unfrequently occurs, we find no such unequivocal evidence of inflammation, the conclusion must be founded on uncertain, if not on inadequate data. The attempt has been made to connect the depositions in the arterial tunics with inflammation. But when we reflect on the period of life at which those deposites usually occur, and on the many changes which all the tissues exhibit in advanced age, we must pause before we can accede to an explanation so sweeping and so exposed to plausible objections.

Dr. Carswell runs over the hypertrophic affections of various parts. The enlargement of the spleen after intermittent fever, would seem to depend on dilatation of the cellulo-vascular structure. The hypertrophy of bone is sometimes great, from inflammation of its own texture or of the periosteum. Hypertrophy of the muscular tissue from irritation is seldom observed, except in the involuntary muscles. Thus the muscles of the bronchi in chronic bronchitis—of the colon in dysentery—of the bladder in stricture, become excessively enlarged.

Dr. Carswell directs attention to the very important practical fact, that after pericarditis, the heart usually enlarges, and the pericardium becomes adherent to its surface. We call this an important practical fact, because, if there be symptoms of affection of the circulating organs, and any history of previous rheumatism, the practitioner should at once suspect the existence of adherent pericardium and hypertrophic heart, and examine that organ, to see if his suspicion is correct.

Enlargement of glandular organs from irritation, and of the various portions of the nervous system from the same cause, is glanced at by our author. There is nothing in his observations on this head to detain us. He admits that hypertrophy sometimes occurs without any obvious cause. Such is the case with some fatty tumors, unnatural increase of the hair, pendulous flaps of integument, and so forth.

An enumeration of the physical characters of hypertrophy concludes the present fasciculus.

The bulk of the part is of course increased in hypertrophy. But, although the volume of the tissue is augmented, the actual size of the whole organ may not be so. Thus, the parietes of the cardiac ventricle, or of the bladder, may be materially increased, yet the heart and bladder may remain unaltered in their general dimensions. The hypertrophy of the walls is then at the expense of the capacity of the cavity. In other instances, there may be hypertrophy of the muscular coat of hollow organs, although the coat is not thicker than natural, nay, even thinner. This is the case when the cavity is much dilated. It is obvious that, in such a case, there must be a greater positive quantity of muscle. Hypertrophy may result from an increase of consistence, and a more compact organization, independently of alterations of volume or capacity. This happens in muscular, cellular, and especially in glandular organs and tissues.

The *weight* of an organ must of course be augmented in hypertrophy. But how are we to determine that the weight is augmented or diminished? What standard of comparison shall we erect? Perhaps, of all organs, the heart is most liable to hypertrophy; and this change in it is of the utmost consequence. Yet we have no generally-admitted standard by which to judge of its enlargement. As Dr. Carswell observes, Laennec considers that the size of the fist may be taken as a standard of comparison for determining the normal dimensions of the heart; Lobstein fixes its weight at nine or ten ounces; Cruveilhier at six or seven ounces; and Bouillaud at eight or nine ounces in persons between twenty-five and sixty years of age, one or two ounces less in persons between sixteen and twenty-five, and in tall and robust persons at ten or eleven ounces.

This discrepancy of opinion respecting the normal weight and dimensions of the heart considered as a whole, appears also in those which have been assigned to the thickness of the walls of its respective cavities. Laennec merely observes that the thickness of the walls of the left ventricle should be a little more than double that of the right. Lobstein gives the following measurements of the normal thickness of the walls of the ventricles and auricles:—right ventricle two lines and a quarter, left ventricle seven lines at its basis, and four lines a little above the apex; right auricle one line, left half a line. Bouillaud fixes the mean thickness of the right ventricle at two lines and a half, and that of the left at seven lines; of the right auricle at one line, and that of the left half a line.

Increase of consistence is the usual concomitant of hypertrophy. It is sometimes considerable in the cellular tissue, lymphatic glands and brain.

The colour of hypertrophied parts is sometimes heightened, but more commonly diminished. The latter probably arises from compression of the bloodvessels *in* the part.

The form of hypertrophied parts is altered, particularly if the hypertrophy be partial. Dr. Carswell remarks, that some of the most marked changes of this description are observed in hollow organs, and he adduces some varieties of dilatation of the bronchi, which deserve attention.

"In the first variety of form which accompanies dilatation, the bronchi of the second, third, or fourth order, present a fusiform shape, each bronchus gradually increasing in size in the direction of its distribution, until it has acquired very considerable dimensions, varying from a quarter of an inch to an inch in diameter. The second variety, which occurs in very small bronchi, and chiefly in those which pass out from the sides of the large bronchi, presents a pyriform shape, the dilated tube having a narrow pediculated attachment, and a rounded fundus from three to six lines in diameter. In the third variety, which is met with generally in bronchi of the fourth order, the dilatation is globular, the whole circumference of these tubes being equally dilated within a circumscribed extent. Sometimes one, but more frequently several globular dilatations, either contiguous to, or separated from each other by irregular intervals, occupy the same bronchus, and vary from a few lines to nearly two inches in diameter. The last variety occurs in the smallest bronchial tubes, several of which being dilated in a circumscribed portion of lung, small groups of round or ovoid bodies, of the size of a hemp-seed, pea, or cherry, are formed, which, when carefully separated from the surrounding pulmonary tissue, present the appearance of a bunch of grapes. This variety is sometimes complicated with emphysema, the dilated bronchi being filled with a thick mucous or muco-purulent secretion, and the air-cells with air, and when not very considerable, may be confounded with the latter disease. It may also, when situated in the upper lobe of the lung, be confounded with a multilocular tubercular excavation, when lined by a smooth mucous membrane of new formation. The globular variety likewise, when it occupies the same situation, is still more likely to give rise to a similar mistake."

Such are the observations of Dr. Carswell on hypertrophy. They are comprehensive and valuable, and deserve the attention of all who would wish to be at the pathological level of the day. The plates of this fasciculus are as beautiful as usual, no inconsiderable merit. We have already observed, in reference to this work, that the library of an English physician or surgeon that wants it is imperfect in a high degree.

We had intended to have introduced into this article a notice of some other pathological works. But we find that it has occupied too much space to permit us to proceed. In our next, we will complete the review of the elements of Mr. Mayo, and lay before our readers a resumé of the latter labours of M. Cruveilhier.

